# Two Distinct Etiologies of Gastric Cancer: Infection and Autoimmunity

**DOI:** 10.3389/fcell.2021.752346

**Published:** 2021-11-26

**Authors:** Stella G. Hoft, Christine N. Noto, Richard J. DiPaolo

**Affiliations:** Department of Molecular Microbiology and Immunology, Saint Louis University School of Medicine, St. Louis, MO, United States

**Keywords:** immunology, gastric cancer, gastric adenocarcinoma, *H. pylori*, AIG = autoimmune gastritis, autoimmunity, gastritis

## Abstract

Gastric cancer is a leading cause of mortality worldwide. The risk of developing gastric adenocarcinoma, which comprises >90% of gastric cancers, is multifactorial, but most associated with *Helicobacter pylori* infection. Autoimmune gastritis is a chronic autoinflammatory syndrome where self-reactive immune cells are activated by gastric epithelial cell autoantigens. This cause of gastritis is more so associated with the development of neuroendocrine tumors. However, in both autoimmune and infection-induced gastritis, high risk metaplastic lesions develop within the gastric mucosa. This warrants concern for carcinogenesis in both inflammatory settings. There are many similarities and differences in disease progression between these two etiologies of chronic gastritis. Both diseases have an increased risk of gastric adenocarcinoma development, but each have their own unique comorbidities. Autoimmune gastritis is a primary cause of pernicious anemia, whereas chronic infection typically causes gastrointestinal ulceration. Both immune responses are driven by T cells, primarily CD4^+^ T cells of the IFN-γ producing, Th1 phenotype. Neutrophilic infiltrates help clear *H. pylori* infection, but neutrophils are not necessarily recruited in the autoimmune setting. There have also been hypotheses that infection with *H. pylori* initiates autoimmune gastritis, but the literature is far from definitive with evidence of infection-independent autoimmune gastric disease. Gastric cancer incidence is increasing among young women in the United States, a population at higher risk of developing autoimmune disease, and *H. pylori* infection rates are falling. Therefore, a better understanding of these two chronic inflammatory diseases is needed to identify their roles in initiating gastric cancer.

## Introduction

Gastric cancer is the fourth leading cause of cancer related mortality with the fifth highest incidence rate worldwide (Hyuna Sung et al., 2021). The discrepancy with lower incidence than mortality rate may be linked to the fact that most gastric cancers are diagnosed late in disease progression due to limited premalignant signs and symptoms. In the United States, late diagnosis contributes to a 5-year survival rate of only 31% for gastric cancer (Matsuzaka et al., 2016). In addition, stomach cancer research has been identified as an underfunded field given its high mortality rate (Carter and Nguyen, 2012). Gastric adenocarcinomas which derive from epithelial cells in the gastric glands make up more than 90% of all gastric cancers. A combination of environmental, host behavior, genetic, and microbial factors contribute to the risk of developing gastric adenocarcinoma (Rawla and Barsouk, 2019). Chronic infection with *Helicobacter pylori* (*Hp*) is recognized as a major risk factor associated with gastric adenocarcinoma development. Autoimmune gastritis (AIG) is another risk factor that is more commonly associated with neuroendocrine tumor development. More thorough investigation is needed into how chronic inflammation, triggered by infection or autoimmunity, causes epithelial cells to undergo premalignant changes that can lead to gastric cancer. The high mortality and poor survival associated with gastric adenocarcinoma make it imperative to better understand disease progression to improve preventative and therapeutic strategies.

### Gastric Cancer Burden in the United States

Within the United States, gastric cancer incidence rates have been decreasing for several decades. However, when incidence is considered by the age at diagnosis (early-onset < 50 years, late-onset >50 years), early-onset incidence has shown a two-fold increase in the last 2 decades (Bergquist et al., 2019). Studies have indicated that this increase in incidence of early-onset individuals is primarily due to an increase in young females (Anderson et al., 2018). Autoimmune disease shows a female-specific predominance and there is a strong possibility AIG follows this trend (Cabrera de León et al., 2012). As well, it has been shown that within the United States the prevalence of *Hp*-positive gastric cancer cases has been continually declining since 2007 and North America is one of the regions with the lowest incidence rates of *Hp* infection (Hooi et al., 2017; Nguyen et al., 2020). Given the low *Hp* infection rates and the specific increase in gastric cancer incidence among young women in the United States, it can be inferred that AIG may be the culprit behind this rise in gastric cancer incidence. Unfortunately, due to underdiagnosis and a lack in research, there is no definitive evidence supporting an increase in AIG incidence among young women over time to confirm this theory (Carmel, 1996; Neumann et al., 2013). However, the incidence rates of autoimmune diseases associated with AIG, autoimmune thyroiditis and type I diabetes, have shown increases in incidence over time (McLeod and Cooper, 2012; Kahaly and Hansen, 2016; Rodriguez-Castro et al., 2018; Mobasseri et al., 2020). As well, benign neuroendocrine tumors in the stomach, associated with AIG, have shown a significant increase in incidence over time and are more common in women (Yao et al., 2008). Combining these facts, it is likely that AIG may also be on the rise and therefore responsible for the increased gastric cancer incidence in young women. It is necessary to learn the pathophysiology behind this etiology of gastric inflammation compared to *Hp* infection to improve early diagnostic and management strategies in patients with a high risk of developing gastric cancer.

### *Helicobacter pylori*-Induced Gastritis

It has been almost 40 years since Barry Marshall and Robin Warren isolated and identified a spiral bacterium present in the stomachs of gastritis patients, now known as *Helicobacter pylori* (*Hp*) (Marshall and Warren, 1984). After personally ingesting a culture of *Hp*, Marshall and others discovered this bacterium could not only colonize the stomach, but also induced an inflammatory response in the epithelium (Marshall et al., 1985; Morris and Nicholson, 1987). Thanks to these pioneers, it is now well established that *Hp* is a Gram negative spirochete capable of chronically colonizing the inhospitable superficial gastric mucosa, leading to diseases like peptic ulceration and chronic gastritis.

**FIGURE 1:**
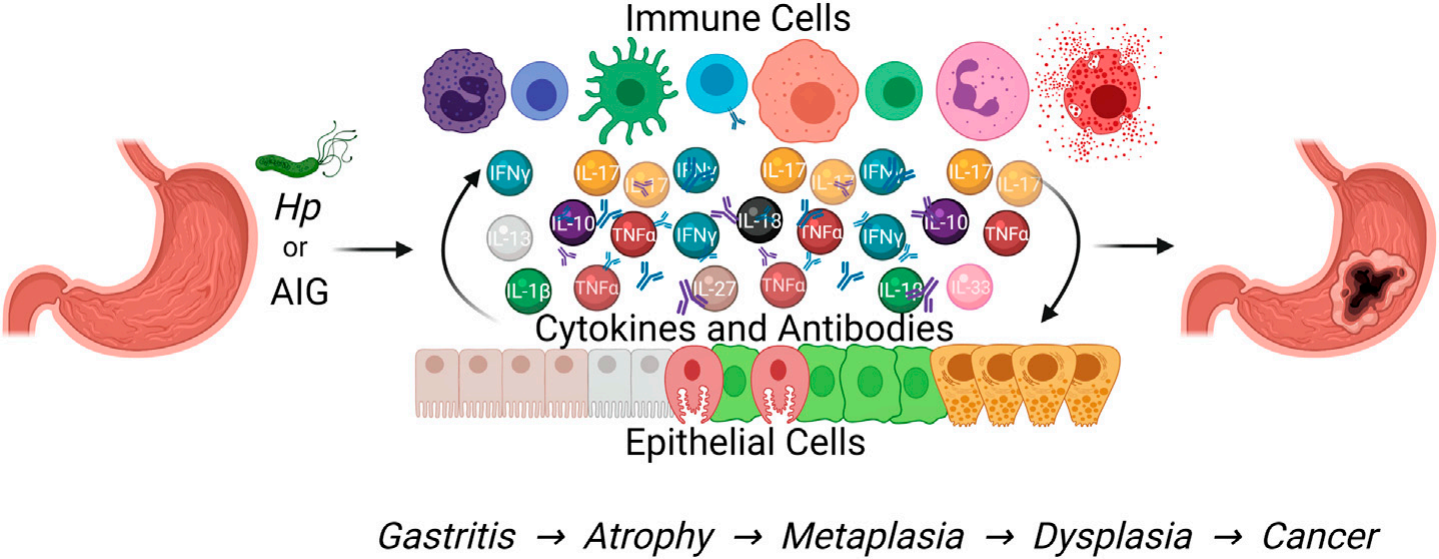
Both *Hp* infection and autoimmune gastritis cause chronic gastric inflammation that can progress to gastric metaplasia and increase the risk of gastric adenocarcinoma development. This Figure was created using BioRender.com.

*Hp* infection has been estimated to contribute to almost 90% of gastric adenocarcinoma cases (Plummer et al., 2015). *Hp* currently infects more than half of the world’s population, however less than 3% of individuals infected with *Hp* will go on to develop gastric adenocarcinoma (Uemura et al., 2001; Hooi et al., 2017). Prevalence is higher than 70% in countries such as Brazil, Nigeria, and Pakistan, and lower than 40% in places such as Switzerland, Australia, and the United States (Hooi et al., 2017) Countries with high prevalence have difficulties controlling infection due to socioeconomic factors like limited access to clean drinking water and health care, especially for children (Malaty and Graham, 1994). Long-term infection with *Hp* can lead to outcomes like dyspepsia, chronic gastritis, peptic ulcer disease, mucosa-associated lymphoid tissue (MALT) lymphoma, gastric metaplasia, and gastric adenocarcinoma (Kusters et al., 2006). Chronic gastritis induced by infection follows a stepwise progression of disease culminating in gastric cancer (Figure 1), originally described as the Correa Cascade (Correa, 1988). Along this pathway, prolonged inflammation leads to oxyntic atrophy: the loss of corpus glands containing acid-secreting parietal cells and digestive enzyme-producing chief cells. Oxyntic atrophy can trigger metaplastic transformations in remaining epithelial cells that over time evolve into dysplasia and cancer (Sáenz and Mills, 2018). Research in vaccination strategies for infection prevention have been underway for decades, but most studies are still in early and preclinical stages of development (Dos Santos Viana et al., 2021). Currently, there are effective *Hp* treatments available as combinations of antibiotics and proton pump inhibitors with/without bismuth subsalicylate (Schistosomes liver flukes and Heclicobacter Pylori, 1994). Downfalls with antibiotics, including resistance, side effects, and inability to prevent reinfection, have promoted research into alternative treatment approaches like probiotics and vaccination, but combination therapy still remains the first line option (Abadi, 2016). Combination therapy administered prior to the development of metaplasia has been reported to reverse progression through the pre-metaplastic cascade (Koulis et al., 2019). Eradication of *Hp* has also been shown to decrease gastric cancer incidence, so clinical guidelines recommend testing for and treating infection in patients with identified gastric lesions (e.g., gastritis, atrophy, metaplasia, dysplasia) followed by confirmation of eradication testing (Lee et al., 2016; Gupta et al., 2020). In fact, confirmed eradication of *Hp* has been found to decrease the recurrence of dysplastic lesions in patients with previously resected gastric neoplasms compared to those who were not treated or failed treatment (Shiotani et al., 2014; Shin et al., 2015). Due to effective treatments and enhanced standard of living, *Hp* infection rates in the United States are low, but other countries around the world still struggle to control the spread of *Hp*.

The chronic gastric inflammation and pathology induced by *Hp* is mediated by immune cell infiltration into the gastric mucosa, producing inflammatory molecules that interact with resident epithelial cells. The cytokines TNFα, IFN-γ, IL-1β, IL-6, IL-8, IL-10, and IL-18 have all shown increased expression measured within gastric biopsies of *Hp-*infected compared to healthy patients (Lindholm et al., 1998; Graham et al., 2004; Moyat and Velin, 2014). There is a prominent lymphocytic infiltrate in *Hp* gastric inflammation, with specific increases in CD4^+^ and CD8^+^ T cells and IgM- and IgG-producing B cells present in the gastric mucosa of *Hp-*infected over uninfected individuals (Nurgalieva et al., 2005). It is typical that the anti-bacterial immune response promotes differentiation of CD4^+^ T cells into Th1 cells that produce IFN-γ and TNFα, as opposed to Th2 cells that produce IL-4 and IL-13 usually in response to parasitic infection or allergy. In fact, it has been established that *Hp*-specific CD4^+^ T cells are predominantly differentiated into Th1 cells in response to *Hp* antigens (e.g., CagA, FlaA, VacA, UreB). IFN-γ, but not IL-4, is reported to be important for promoting gastric inflammation (Nurgalieva et al., 2005; Smythies et al., 2000; D'Elios et al., 1997). Some hypothesize that early parasitic infection skews an individual’s immune response toward Th2, decreasing the risk of *Hp* associated gastric cancer; however others have found increased levels of the type 2 cytokine, IL-13, in chronically *Hp* infected individuals to be associated with enhanced disease progression beyond inflammation (Holcombe, 1992; Marotti et al., 2008). Therefore, it is likely that the immune response to *Hp* is heterogenous, but the early inflammatory response is skewed Th1 over Th2. Recent studies have shown that both Th1 and Th17 (IL-17 producing) cells play important roles in the induced response against infection. One study found that Th17 cells precede Th1 and are important for inducing the Th1 response (Shi et al., 2010). IL-17 also plays a role in recruiting neutrophils to the site of infection (DeLyria et al., 2009). In mice infected with *Helicobacter* species, neutrophil depletion slowed clearance and limited inflammation-mediated pathology (Ismail et al., 2003; DeLyria et al., 2009). A proposed mechanism of neutrophil-induced pathology is *Hp* phagosome escape strategies causing reactive oxygen species to leak into the extracellular environment and elicit tissue damage (Allen et al., 2005). Other cells, including eosinophils and mast cells, have also been identified within the gastric mucosa of *Hp*-infected individuals, although their exact contributions to disease progression and/or infection clearance remains unclear (Nakajima et al., 1997; Moorchung et al., 2006).

Antigen-presenting cells are important for *Hp-*mediated inflammation through the activation of autoreactive CD4^+^ T cells. Dendritic cells presenting *Hp* antigen have been found to stimulate CD4^+^ T cells *in vitro* to produce IFN-γ, TNFα, and IL-17 (Hafsi et al., 2004; Khamri et al., 2010). In contrast, classical dendritic cells expressing Programmed Death Ligand 1 (PDL1) were recently found in the gastric submucosa of *Hp*-infected individuals interacting with effector T cells to promote tolerance and limit gastric pathology (Go et al., 2021). Epithelial cells within the inflamed gastric tissue may also be responsible for lymphocyte activation. Studies have demonstrated that molecules like HLA-DR (MHC II), ICAM-1 (lymphocytic adhesion), B7-1 and B7-2 (T cell co-stimulation), and Fas/FasL (receptor mediated apoptosis) have increased expression on epithelial cells in *Hp*-infected tissue compared to healthy controls (Ye et al., 1997; Archimandritis et al., 2000; Wang et al., 2000). These molecules contribute to immune cell-mediated epithelial cell apoptosis and epithelial cell-mediated T cell proliferation and activation. In summary, infection with *Hp* causes an infiltration of immune cells, which leads to chronic inflammation and inflammation-mediated pathology. A more in-depth understanding of the specific immune response influencing the risk of gastric cancer is still needed.

### Autoimmune Gastritis

Autoimmune gastritis (AIG) is the consequence of an individual’s immune system lacking appropriate tolerance to self-antigens. This allows autoreactive T cells to become activated against gastric parietal cells, leading to chronic gastritis and oxyntic atrophy. Due to inefficient tolerance by the immune system to self-antigens, AIG can occur as part of multiple autoimmune syndrome, in which a person is diagnosed with three or more distinct autoimmune diseases (Cojocaru et al., 2010). Commonly, these individuals have diseases like autoimmune thyroiditis and type one diabetes mellitus alongside autoimmune gastritis (Kahaly and Hansen, 2016; Rodriguez-Castro et al., 2018). Immune destruction of parietal cells in AIG can cause a loss of intrinsic factor, a protein produced by parietal cells required for Vitamin B12 absorption in humans. The resulting megaloblastic anemia due to Vitamin B12 deficiency is referred to as pernicious anemia (PA) (Toh et al., 1997). Since Vitamin B12 has long term storage in the liver, an absorptive deficiency may go undetected for years, allowing time for gastric inflammation, atrophy, and metaplasia to develop, as shown in Figure 1 (Lenti et al., 2019). Patients diagnosed with PA require lifelong Vitamin B12 supplementation to prevent severe clinical manifestations of deficiency (Green et al., 2017). Another outcome from the loss of acid-secreting parietal cells is hypochlorhydria. Increased gastric pH enhances gastrin production from G cells in the stomach. Gastrin acts on and induces hyperplasia of neuroendocrine ECL cells, which promote acid secretion from parietal cells (Walsh, 1990). This type of hyperplasia is a common feature of AIG and can lead to the development of generally benign neuroendocrine tumors (Müller et al., 1987; Coati et al., 2015). The combination of the insidious nature of PA’s disease progression, the accepted association of AIG with neuroendocrine tumors, and a focus on *Hp* as the major risk factor for gastric adenocarcinoma may have contributed to a lack of appreciation for the gastric cancer risk associated with AIG. However, recent studies have shown that AIG patients have a significantly increased risk of developing gastric cancer (Landgren et al., 2011; Hemminki et al., 2012). In one such study, Vannella et al. estimates that patients suffering from AIG have a seven-fold increased relative risk which is equivalent to the relative risk associated with *Hp* infection (Kamangar et al., 2006; Vannella et al., 2013).

It is well-established that the primary etiology of autoimmune gastritis is the targeted atrophy of Parietal cells via the activation of parietal cell-specific T and B cells in the stomach. Antibodies against parietal cells and intrinsic factor can be found in the serum of most AIG patients (Taylor et al., 1962; Rusak et al., 2016). Autoreactive CD4^+^ T cells mainly target the H^+^/K^+^ ATPase proton pump found on parietal cells and predominantly differentiate into the Th1 phenotype, producing IFN-γ upon stimulation with this antigen (D'Elios et al., 2001). These Th1 cells are also capable of inducing IgM, IgG and IgA production from autologous B cells *in vitro* and can induce pathology via perforin/granzyme or Fas/FasL-mediated cell death. Dendritic cells (DC) have also been identified in gastric biopsies from AIG patients and in mice an increase in gastric DCs correlated to enhanced pathology (Ninomiya et al., 2000). In a prospective study, women who developed AIG were found to have T cell and macrophage infiltration in their stomach biopsies as well as increased HLA-DR (MHC II) expression on epithelial cells (Burman et al., 1992). Mast cells and eosinophils have also been identified in the setting of AIG, but their contribution to disease and the significance of infiltration have not yet been determined (Park et al., 2013; Bockerstett et al., 2020).

There is limited data addressing the specific immune cell responses leading to autoimmune gastritis in humans, however there are experimental models that have expanded upon this topic. Neonatal thymectomy of mice can induce autoimmune gastritis amongst other autoimmune diseases. In this model, the thymus is removed prior to day three of life, preventing the development of regulatory T cells (Tregs) that promote tolerance and limit autoimmunity (Suri-Payer et al., 1998; Fontenot et al., 2005). In these animals autoreactive T cells escape tolerance and enter the periphery to be activated by self-antigens. The gastritis in these thymectomized mice is caused by parietal cell specific CD4^+^ T cells infiltrating the gastric mucosa and initiating disease, followed by autoreactive B cells producing anti-parietal cell antibodies (Fukuma et al., 1988).

McHugh et al. isolated CD4^+^ T cells from neonatally thymectomized mice that developed AIG and cloned a T cell receptor (TCR) specific for a peptide from the parietal cell H^+^/K^+^ ATPase α chain, the major autoantigen in both mice and humans. This TCR sequence was used to generate a CD4^+^ TCR transgenic mouse model of AIG referred to as the TxA23 mouse (McHugh et al., 2001). In this model, the transgenic CD4^+^ T cells initiate disease progression which follows the same pattern seen in humans: gastritis progressing to atrophic gastritis by 2 months of age, metaplasia by 4 months of age, and then mice begin to show signs of gastric dysplasia by 12 months of age (McHugh et al., 2001; Nguyen and Dipaolo, 2013). TxA23 mice generate an adaptive immune response against parietal cells similar to humans, producing parietal cell autoantibodies and similar inflammatory cytokines like IFN-γ and IL-17 (Nguyen et al., 2013). IL-17 and IFN-γ in this model have been found to induce parietal cell atrophy initiating pathology in the corpus of the stomach (Bockerstett et al., 2018; Osaki et al., 2019). The type two cytokine IL-13 was also found to have a profound impact on progressing gastric pathology beyond atrophic gastritis in TxA23 animals, driving metaplastic transformation in the tissue (Noto et al., 2021). These mice also show significant increases in pSTAT3 and IL-6, two molecules known to be associated with human carcinomas (Nguyen et al., 2013). IL-27, produced by macrophages in the gastric mucosa, is protective against inflammation and metaplastic development by acting on CD4^+^ T cells to dampen the inflammatory response (Bockerstett et al., 2020). Although these and other recent studies of immune responses during autoimmune gastritis have increased our understanding of the pathogenesis of AIG, the progression from gastritis to gastric cancer in AIG is still incompletely understood.

### Comparison of *Helicobacter pylori* and Autoimmune Gastritis

There are established similarities and differences between AIG and *Hp*-induced gastritis which are summarized in Table 1. As previously stated, both *Hp* and AIG cause inflammation-mediated pathology that can lead to the appearance of metaplastic gastric glands, chronic gastritis, parietal cell atrophy, and eventually gastric adenocarcinoma. Prominent unique late manifestations are pernicious anemia, ECL cell hyperplasia, and neuroendocrine tumors in AIG and peptic ulceration and MALT lymphoma in *Hp*. Iron deficiency anemia, a microcytic anemia, can emerge early on in both settings, but pernicious anemia, a macrocytic anemia, is primarily associated with late-stage AIG (Park et al., 2013; Amieva and Peek, 2016; Kulnigg-Dabsch, 2016). Surprisingly, studies have found that 92% of patients with MALT lymphoma, a primary B cell lymphoma, are infected with *Hp*, but no strong correlation between this type of lymphoma and AIG has been identified (Wotherspoon et al., 1991). In AIG, the targeted loss of acid-secreting parietal cells causes a rise in gastric pH and increased gastrin production, triggering hyperplasia of acid-promoting neuroendocrine cells from which neuroendocrine tumors can arise (Walsh, 1990; Park et al., 2013). Measured gastric pH can be high, low, or normal in *Hp* patients depending on the localization, length of infection, and extent of inflammation and atrophy, but there is no strong association between *Hp* infection and neuroendocrine tumor development (McColl et al., 1998). Differences in extent of parietal cell atrophy and impact on gastric pH probably contribute to the divergence in clinical manifestations, driving prominent ECL cell hyperplasia and neuroendocrine tumor development in AIG but not *Hp.* AIG initiates and remains restricted to the corpus of the stomach primarily targeting parietal cells, whereas *Hp* initially colonizes the antrum and spreads through to the corpus after long-standing infection, causing a patchy or multifocal disease (Graham et al., 2019).

**TABLE 1:** A visual overview comparing infection and autoimmunity induced gastritis.

	*H. pylori-Induced* Gastritis	Autoimmune Gastritis
Clinical Manifestations	Peptic Ulcer Disease	Pernicious Anemia
	Mucosa-Associated Lymphoid Tissue Lymphoma	ECL Hyperplasia/Neuroendocrine Cell Tumor
	Iron Deficiency Anemia	Multiple Autoimmune Syndrome
	Gastric Metaplasia, Dysplasia, Adenocarcinoma	Iron Deficiency Anemia
		Gastric Metaplasia, Dysplasia, Adenocarcinoma
Localization	Initially colonizes the antrum and then spreads to the corpus causing a multifocal disease	Remains restricted to the corpus region
Elevated Serum Values	Anti-Hp IgG	Anti-Parietal Cell/Intrinsic Factor Antibodies
	Occasionally Gastric Autoreactive Antibodies	Occasionally Anti-Hp lgG
		Gastrin
Lymphocyte Targets	Various Hp Antigens (e.g., CagA, FlaA, VacA, UreB)	Parietal Cells
		Intrinsic Factor
Inflammatory Infiltrates	Adaptive: Th1 and Th17 Cells, B Cells	Adaptive: Th1 and Th17 Cells, B Cells
	Innate: Neutrophils, Macrophages, Dendritic Cells, Mast Cells, Eosinophils	Innate: Macrophages, Dendritic Cells, Mast Cells, Eosinophils
	Epithelial Cells Become Antigen Presenting Cells	Epithelial Cells Become Antigen Presenting Cells
Management and Surveillance	Eradication Therapy	Vitamin B12 Supplementation
	Routine Endoscopic Screening Dependent on Presence of Intestinal Metaplasia, Risk Factors, Patient Preference, Extent of Disease	Routine Endoscopic Screening Dependent on Presence of Intestinal Metaplasia, Risk Factors, Patient Preference, Extent of Disease

As previously detailed, the immune responses triggered by AIG and *Hp* in the gastric mucosa have prominent similarities with some notable variance, but directed studies are needed to establish where these immune responses diverge. Remarkable similarities include a considerable lymphocytic infiltration with predominant Th1- and Th17-differentiated effectors and inflammation-responding epithelial cells upregulating MHC II molecules to aid in CD4^+^ T cell activation (Smythies et al., 2000; Shi et al., 2010; Archimandritis et al., 2000; D'Elios et al., 2001; Burman et al., 1992; Nguyen et al., 2013). While type two cytokines, IL-4 and IL-13, are not responsible for initiating inflammation in response to either *Hp* or AIG, studies have found them to be critical for promoting severe metaplastic and even dysplastic lesions as disease progresses beyond gastritis (Marotti et al., 2008; Gabitass et al., 2011; Miska et al., 2018; Petersen et al., 2018; De Salvo et al., 2021; Noto et al., 2021). Cells like dendritic cells, macrophages, mast cells, and eosinophils have also been identified in both disease settings (Burman et al., 1992; Nakajima et al., 1997; Ninomiya et al., 2000; Suzuki et al., 2002; Hafsi et al., 2004; Moorchung et al., 2006; Khamri et al., 2010; Park et al., 2013; Bockerstett et al., 2020; Go et al., 2021). Key immunological differences include an abundance of autoantibodies in the serum of AIG patients and a clear neutrophilic gastric infiltrate in *Hp* infection important for inducing pathology and clearance (Taylor et al., 1962; Ismail et al., 2003; DeLyria et al., 2009; Rusak et al., 2016). ROS producing neutrophils may be responsible for inducing peptic ulceration in *Hp* but not AIG. Autoimmune gastritis directly targets parietal cells and induces the production of parietal cell and intrinsic factor autoantibodies, while in *Hp* various anti-*Hp* IgGs can be detected in the serum (Taylor et al., 1962; Pan et al., 2014). In some *Hp-*infected patients, autoreactive antibodies can also be detected (Faller et al., 1997; Ayesh et al., 2013). In comparing the immunologic responses, several factors distinguish or relate AIG- and *Hp*-mediated inflammation.

Current gastric precancerous diagnostics use the severity and extent of inflammation, atrophy, and metaplasia present in both the antrum and corpus to grade and stage disease progression, placing the emphasis for cancer risk on gastric pathology, as opposed to etiology of disease. This equates the risk of adenocarcinoma development attributed to AIG and *Hp* once metaplastic lesions arise (Rugge and Genta, 2005; Rugge et al., 2016). However, there is ongoing debate about how frequently, if at all, patients with metaplastic lesions should be routinely surveyed (Pimentel-Nunes et al., 2019; Gupta et al., 2020). For patients in the United States with a late disease stage and compounding risk factors (e.g., racial/ethnic minorities, immigrants from high incidence countries, family history) guidelines suggest the decision for routine endoscopic screening be left up to the patient. Controversy surrounds these guidelines since other premalignant lesions with similar associated cancer risk, like in the esophagus, have stricter surveillance recommendations (Huang and Hwang, 2020). In other countries, such as the United Kingdom, routine endoscopic screening is recommended every 3 years in patients with extensive premalignant lesions and even retroactively in healthy individuals at increased risk of gastric cancer development (Banks et al., 2019). In high incidence countries like Japan and South Korea national gastric screening programs exist (Rawla and Barsouk, 2019). While screening guidelines and practices vary greatly throughout the world, it is widely accepted that gastric metaplasia is a risk factor for gastric adenocarcinoma. Overall, these identified differences and similarities between AIG and *Hp*-induced gastritis are crucial and require further investigation to fully understand the progression to gastric adenocarcinoma arising from either disease setting.

### *Helicobacter pylori* Inducing Autoimmune Gastritis

There are studies suggesting that infection with *Hp* can trigger an autoimmune response within the gastric mucosa. Around 65% of *Hp*-infected individuals have detectable levels of autoreactive gastric antibodies (Negrini et al., 1996). These autoantibodies were most frequently specific to parietal cells and an increase in autoantibodies positively correlated with gastric disease severity (Negrini et al., 1996; Faller et al., 1997). *Hp*-infected people with serum-detectable gastric autoantibodies showed increased corpus atrophy, decreased stomach acid production, and increases in routine AIG diagnostic markers. In addition to AIG, studies have found *Hp* infection to be associated with a variety of other autoimmune diseases (Smyk et al., 2014).

Molecular mimicry has been suggested as a potential mechanism for developing autoimmunity out of infection. A major *Hp* surface protein, β urease, has 72% sequence homology with the β chain of the parietal cell specific H^+^/K^+^ ATPase and has been suggested as one possible antigen activating *Hp* effector cells against gastric tissue (Uibo et al., 1995). In *Hp* infected patients who developed AIG, CD4^+^ T cells were isolated from gastric biopsies, cloned, and stimulated in the presence of H^+^/K^+^ ATPase peptides or *Hp* peptides from lysate (Amedei et al., 2003). Several clones were cross-reactive against peptides from parietal cells and *Hp* lysate and stimulation in the presence of both antigens induced T cells to produce large and equivalent amounts of IFN-γ (Smythies et al., 2000; D'Elios et al., 1997; D'Elios et al., 2001). These cross-reactive cells were differentiated into the Th1 cells typically present in both AIG and *Hp*-induced gastritis. Also, in *Hp* infection, it has been shown that epithelial cells increase antigen presentation capabilities by upregulating MHC II molecules on their surface, increasing the possibility for self-peptide presentation (Archimandritis et al., 2000). This could be another way to induce autoreactive cells without the need for molecular mimicry.

Overall, worldwide prevalence of *Hp* infection is around 50%, but this frequency can range between 40–60% depending on age, social class, and geographic region, among other factors (Sitas et al., 1991; Hooi et al., 2017; Wang et al., 2019). Around 60% of pernicious anemia (PA) patients have serologic and/or histologic evidence of *Hp* infection, close to the frequency found in the general population (Annibale et al., 2000; Presotto et al., 2003). This infection prevalence among PA patients does not show a correlation between *Hp* infection and PA. Other studies have shown that PA patients have a lower frequency of *Hp* positivity than the general population. Some speculate that atrophy endured from AIG may deter *Hp* colonization as even fewer PA patients with severe gastric body atrophy show evidence of infection (Pérez-Pérez, 1997; Presotto et al., 2003). However, a recent study by Saenz et al. showed that in *Hp*-infected mice an atrophy-induced increase in gastric pH promotes *Hp* corpus colonization and that *Hp* preferentially binds deeper within antralized metaplastic corpus glands (Sáenz et al., 2019). This may make it more difficult to detect *Hp* in severely diseased, shallow human biopsy samples. It has also been shown that *Hp*-infected individuals can have seroconversion, or the loss of detectable serologic anti-*Hp* immunoglobulin after years of infection with and without successful eradication therapy (Valle et al., 1996; Perez-Perez et al., 2002; Veijola et al., 2007). Severe gastritis leading to clearance or deep binding of bacteria, combined with a loss of serologic positivity, could explain why PA patients don’t show higher rates of *Hp*, although these ideas still do not prove that AIG is triggered by infection. A decrease in *Hp* prevalence among the United States population and a rise in gastric cancer incidence in young American females possibly points to the independent development of autoimmune gastritis (Anderson et al., 2018; Bergquist et al., 2019). There has been clear documentation of AIG patients without evidence of *Hp* infection that typically have comorbidities with other autoimmune diseases and have unique diagnostic criteria more associated with *Hp*-negative AIG (Venerito et al., 2016). To date, there have been no studies definitively showing that *Hp* induces AIG, and therefore no confident conclusions can yet be drawn.

Further experimentation is needed to prove a link between *Hp* and AIG. To definitively establish that *Hp* infection induces AIG, a prospective study would need to be conducted on chronically *Hp* infected patients. Patients would get sequential blood draws to measure if/when gastric autoantibodies can be detected in the serum. Finding patients for this study would be extremely difficult considering they would need to be untreated or treatment resistant. This would require a multi-national approach which would be logistically very difficult and expensive to perform. In the animal model it would be feasible to infect mice and serially measure serum for the emergence of autoantibodies, but this would not definitely establish the link in humans. It is also a challenge to establish the significance of these studies, as the results would not necessarily change anything about patient management. Overall a combination of high expense, low significance, and difficultly conducting the experiments may leave the question of whether *Hp* induces AIG unanswered.

## Conclusion

Gastric cancer is a world leading cause of cancer related mortality (Hyuna Sung et al., 2021). It has been well-established that gastric infection with *Hp* is the major risk factor for developing gastric cancer. This bacterium still infects a large portion of the world population, but infection rates in the United States are low and incidence of *Hp*-negative gastric cancer is increasing (Hooi et al., 2017; Nguyen et al., 2020). As well in the United States, gastric cancer incidence rates are increasing specifically among young females (Hooi et al., 2017; Anderson et al., 2018; Bergquist et al., 2019). These trends converge to the likelihood that United States gastric cancer may arise out of AIG. While no definitive evidence exists for a rise in AIG incidence to corroborate this hypothesis, increases in related diseases (type I diabetes, autoimmune thyroiditis, gastric neuroendocrine tumors) suggest this may be the case (Yao et al., 2008; McLeod and Cooper, 2012; Kahaly and Hansen, 2016; Rodriguez-Castro et al., 2018; Mobasseri et al., 2020). In the past, AIG has been missed as a significant risk factor for gastric cancer, but the risk associated with AIG may be just as high as with *Hp* infection and the major pathology-inducing immune cells are consistent in both disease settings (Smythies et al., 2000; D'Elios et al., 1997; Vannella et al., 2013; D'Elios et al., 2001). Some unanswered questions between these two etiologies remain. For example, it is not definitively known why neuroendocrine tumors do not arise out of *Hp* infection and peptic ulcers do not develop in AIG patients. Differences in the extent of parietal cell atrophy and subsets of infiltrating immune cells may contribute to these divergent manifestations. Speculation on whether the immune response to *Hp* infection can induce autoimmune gastritis is based on correlative studies, but there is no definitive evidence to prove that *Hp* infection initiates the autoimmune response found in AIG. Studies needed to prove this link would be challenging and costly. This review aims to highlight two risk factors for gastric cancer, *Hp* and AIG, and compile what is currently in the literature for how these inflammatory triggers lead to gastric cancer. Further immunological studies on the progression from healthy tissue through inflammation to cancer is required for these two etiologies of gastritis to diminish the global burden of gastric cancer.
